# Disease-Associated Neurotoxic Astrocyte Markers in Alzheimer Disease Based on Integrative Single-Nucleus RNA Sequencing

**DOI:** 10.1007/s10571-024-01453-w

**Published:** 2024-02-12

**Authors:** Wuhan Yu, Yin Li, Fuxin Zhong, Zhangjing Deng, Jiani Wu, Weihua Yu, Yang Lü

**Affiliations:** 1 Department of Geriatrics, The First Affiliated Hospital of Chongqing Medical University, No.1 Youyi Road, Yuzhong, Chongqing, 400016 China; 2 Department of Thoracic Surgery, Zhongshan Hospital, Fudan University, Shanghai, 200032 China; 3 Institutes of Neuroscience, Chongqing Medical University, Chongqing, 400016 China

**Keywords:** Alzheimer disease, Biomarkers, Neurotoxic astrocytes, Bulk RNA-seq, snRNA-seq

## Abstract

Alzheimer disease (AD) is an irreversible neurodegenerative disease, and astrocytes play a key role in its onset and progression. The aim of this study is to analyze the characteristics of neurotoxic astrocytes and identify novel molecular targets for slowing down the progression of AD. Single-nucleus RNA sequencing (snRNA-seq) data were analyzed from various AD cohorts comprising about 210,654 cells from 53 brain tissue. By integrating snRNA-seq data with bulk RNA-seq data, crucial astrocyte types and genes associated with the prognosis of patients with AD were identified. The expression of neurotoxic astrocyte markers was validated using 5 × FAD and wild-type (WT) mouse models, combined with experiments such as western blot, quantitative real-time PCR (qRT-PCR), and immunofluorescence. A group of neurotoxic astrocytes closely related to AD pathology was identified, which were involved in inflammatory responses and pathways related to neuron survival. Combining snRNA and bulk tissue data, *ZEP36L, AEBP1, WWTR1, PHYHD1, DST* and *RASL12* were identified as toxic astrocyte markers closely related to disease severity, significantly elevated in brain tissues of 5 × FAD mice and primary astrocytes treated with Aβ. Among them, WWTR1 was significantly increased in astrocytes of 5 × FAD mice, driving astrocyte inflammatory responses, and has been identified as an important marker of neurotoxic astrocytes. snRNA-seq analysis reveals the biological functions of neurotoxic astrocytes. Six genes related to AD pathology were identified and validated, among which WWTR1 may be a novel marker of neurotoxic astrocytes.

**Supplementary Information:**

The online version contains supplementary material available at 10.1007/s10571-024-01453-w.

## Introduction

Alzheimer disease (AD) is a destructive neurodegenerative disorder characterized by the gradual decline in cognitive and physical functions (Scheltens et al. 2021). It is a leading cause of impaired self-care ability in the elderly. There is a lack of effective treatments, resulting in a significant impact on the patients’ quality of life and causing substantial suffering for both patients and their families (Jia et al. 2018). Moreover, with advances in modern medicine expanding the average human lifespan, the current worldwide incidence of dementia is more than 50 million people and is expected to treble in the next 30 years (Grimaldi et al. 2019; Jia et al. 2020). The increasing number of AD patients yearly will become a heavy burden on the public health system (Wong 2020). Therefore, it is urgent to identify the key target molecules and biomarkers for reversing its pathological progression. However, classical research focused on neurons has several limitations. Consequently, an increasing number of researchers are turning their attention to the role of glial cells in AD (Uddin and Lim 2022).

Glia play a crucial role in disease mitigation, whereas glial dysfunction contributes to brain disorders (Barres 2008). Astrocytes are the most abundant type of glial cells in the brain, and their population significantly outnumbers neurons (Verkhratsky and Nedergaard 2018). In the context of AD, astrocytes are involved in both beneficial and detrimental processes. Normal astrocytes can regulate neurotransmitter and calcium homeostasis, modulate synapse formation, maturation, and elimination, regulate blood–brain barrier function through neuron-glia-vascular units, control extracellular space volume and ion homeostasis, and provide nutritional and trophic support to the brain (Ferrer 2017). However, the neurotoxic and pro-inflammatory phenotype of A1-like astrocytes likely contributes to the progression of AD pathology (Liddelow et al. 2017). A1 astrocytes increase the production of inflammatory cytokines and lose phagocytic activity, ultimately showing a compromised ability to effectively clear amyloid-beta (Aβ) aggregates (Lawrence et al. 2023). They also exhibit a loss of their normal supportive functions for synapses and contribute to synaptic dysfunction (Balu et al. 2019). Understanding the mechanisms underlying the transition to the A1-like reactive state and developing strategies to modulate or reverse this phenotype is an active area of research for potential therapeutic interventions. Identifying biomarkers that reflect the pathological state of astrocytes in AD is also of paramount importance.

Single-nucleus RNA sequencing (snRNA-Seq) technologies have advanced rapidly in recent years, especially in the field of neurodegeneration diseases. SnRNA-Seq allows the identification and characterization of distinct cell types within the brain, including neurons, astrocytes, microglia, oligodendrocytes, and endothelial cells (Wang et al. 2022; Mathys et al. 2019). By examining gene expression profiles of individual cells, researchers can identify specific cell populations affected by AD and investigate how they contribute to disease progression (Pandey et al. 2022). Therefore, utilizing snRNA-seq technology to analyze the characteristics of disease-associated neurotoxic astrocytes is an important direction in AD research.

In summary, the neurotoxic reactive astrocytes may drive the pathological progression of AD through various mechanisms, but their functional characteristics and markers remain unclear. SnRNA-Seq has emerged as a powerful technique for investigating cellular heterogeneity and identifying distinct cell populations. Therefore, the aim of the current study is to employ an integrative approach, combining snRNA-Seq analysis with multiple experimental methods, to elucidate the functional characteristics and identify specific markers associated with neurotoxic reactive astrocytes in AD.

Here, three snRNA-Seq datasets of human brain tissues were integrated, and then cell clustering and definition on the merged data were performed. Subsequently, the data of astrocytes were extracted for dimensionality reduction. Cell clusters of disease-associated astrocytes (DAAs) and A1 subtypes were identified. Then the dysregulated pathways related to these cell clusters were explored. By combining these findings with AD-related differentially expressed genes from Bulk RNA-sequencing data of brain tissue, twelve key differentially expressed genes associated with astrocytes in AD patients were eventually identified and the clinical relevance was also analyzed. Finally, the expression of six differentially expressed genes significantly correlated with disease progression were validated in 5 × FAD mice and primary astrocytes. These findings provide new insights into the role of astrocytes in AD.

## Methods

### Single-Nucleus RNA Sequencing Data Collection and Processing

A targeted search was conducted on PubMed for research articles until 2022 that contained the terms 'Alzheimer's disease' and 'Single-nucleus RNA sequencing'. Each article retrieved was subjected to an in-depth review. Our selection criteria were as follows: 1) Studies must use human brain tissue samples without any pre-selection for specific cell populations; 2) The sample size should be greater than 10; 3) The study must include both AD and control groups; 4) The research should be supported by peer-reviewed published literature. Finally, three snRNA-seq datasets of AD were included in this study, including the data GSE138852 (Grubman et al. 2019), GSE157827 (Lau et al. 2020) and GSE174367 (Morabito et al. 2021) (Table 1).

**Table 1:** Dataset characteristics

Dataset	Platform/technolog	No. of samples	Sample source	Age	Gender female:male	Country	Data type
GSE138852	GPL18573 [Illumina NextSeq 500 (Homo sapiens)]	12 (6 AD, 6 controls)	Entorhinal cortex	AD:(67–91y); control(67–90y)	4:8	Singapore	SnRNA-seq
GSE157827	GPL24676 [Illumina NovaSeq 6000 (Homo sapiens)]	21 (12 AD, 9 controls)	Prefrotnal cortex	AD:(60–95y); control(74–94y)	7:14	China	SnRNA-seq
GSE174367	GPL24676 [Illumina NovaSeq 6000 (Homo sapiens)]	20 (12 AD, 8 controls)	Prefrotnal cortex	AD:(81–90y); control(74–90y)	9:11	USA	SnRNA-seq
GSE33000	GPL4372 (Rosetta/Merck Human 44 k 1.1 microarray)	467 (310 AD, 157 controls)	Prefrotnal cortex	AD:(53–100y); control(22–106y)	209:258	USA	Bulk RNA-seq
GSE48350	GPL570 [(HG-U133_Plus_2) Affymetrix Human Genome U133 Plus 2.0 Array]	253 (80 AD, 173 controls)	Hippocampus, entorhinal cortex,superior frontal cortex, post-central gyrus	AD:(60–95 y); control(20–99y)	129:124	USA	Bulk RNA-seq
GSE106241	GPL24170 [Agilent-044312 Human 8 × 60 K Custom Exon array (Probe Name version)]	60 (60 AD)	Inferior termporal cortex	AD:50–100 y	42:18	Finland	Bulk RNA-seq

The dataset GSE138852 included postmortem entorhinal cortex (EC) samples from six AD patients and six sex- and age-matched controls. The GSE157827 dataset comprised 21 prefrontal cortex (PFC) tissue samples, including 12 from AD patients and 9 from control subjects. The GSE174367 included PFC data from 20 postmortem brains, with 12 cases diagnosed with AD. A total of 53 individuals were included in the study, comprising 20 females and 33 males. Among the AD patients, the age ranged from 60 to 95 years, while the control group had an age range of 67 to 94 years.

The processed data of each dataset was separately loaded into R using Seurat (version 4.0.5). The quality of cells was assessed based on five metrics: (1) the number of total UMI count per cell (library size) was below 30,000; (2) the number of detected genes was above 500 and below 6000; (3) the percentage of mitochondrial genes was below 50%. (4) Cells with > 200 genes detected were selected for further analyses. And (5) cells with > 20% of transcripts derived from mitochondria were considered apoptotic, and thus excluded. Then, doublets were removed using DoubletFinder with default settings for each sample.

After discarding low-quality cells, the data was processed using the Harmony package in R for data integration with default parameters. The integration involved aligning cells from different batches or experimental conditions based on the "orig.ident" variable. The convergence plot was utilized to visualize the convergence during the Harmony algorithm's execution. The k-means algorithm was initialized with 20 initial cluster assignments and allowed for a maximum of 10,000 iterations to converge. The Harmony algorithm itself was set to iterate a maximum of 200 times for convergence. Following the completion of the Harmony run, further dimensionality reduction and clustering were performed on the integrated data to identify cellular subpopulations. Cell clusters in each sample were identified by examining the top 30 principal components (PCs) across highly variable genes (HVGs). The markers for each cell cluster were identified using the Seurat 'FindAllMarkers' function, and singleR was applied for cell cluster annotations. To categorize the major cell lineages, a set of marker genes based on previous studies was utilized. For astrocytes, markers included *GFAP, FGFR3, GJA1, AQP4, ALDH1L1;* for excitatory neurons, *SYN3, RBFOX3, CAMK2A*; for inhibitory neurons, *ERBB4, NXPH1, GAD1, GAD2*; for microglia, *HLA-DRA, CX3CR1, C1QB, CSF1R*; for oligodendrocytes, *MOBP, MBP, PLP1*; for oligodendrocyte progenitor cells (OPCs), *PCDH15, MEGF11*; and for endothelial cells, *FLT1, CLDN5* (Chen et al. 2023; Darmanis et al. 2015).

After determining the major cell lineages, subclustering of astrocytes was conducted using Seurat. Different resolution parameters (from 0.1 to 2.0) were tested. For each parameter, the marker genes (Log fold change > 0.25 and adjusted p-value < 0.05) of each subcluster were checked. To obtain as many subclusters as possible, the highest resolution parameter was chosen when the marker genes of any subcluster showed less than 30% overlap with those of any other subclusters. Additionally, some manual adjustments were made to avoid over-clustering. Signature scores were then computed using the Seurat 'AddModuleScore' function, utilizing the gene signature of interest (DAAs marker and A1-specific transcripts) to identify the neurotoxic astrocytes subcluster.

### Gene Ontology (GO) Enrichment and Kyoto Encyclopedia of Genes and Genomes (KEGG) Pathway Analysis of the Subcluster-Specific Genes

GO enrichment analyses were performed in R using the clusterProfiler package. Metascape was used to perform the KEGG pathway analysis. Functional and pathway enrichment analyses were conducted for subcluster-specific genes of neurotoxic astrocytes. In this analysis, an adjusted p-value < 0.05 was considered significant for the screening of significant GO terms.

### Bulk RNA-Sequencing Data Collection and Co-Differentially Expressed Genes Identification

Bulk RNA-sequencing data of AD patients with complete follow-up information were collected from Gene Expression Omnibus (GEO) databases. According to the following criteria, datasets were considered eligible for our analysis: (1) datasets with AD samples; (2) datasets supported by peer-reviewed PubMed-indexed publications; (3) datasets with brain tissue from the EC or PFC. Two datasets related to AD, GSE33000 and GSE48350, were selected for co-differentially expressed genes analysis. Each dataset was isolated to minimize batch effects and inter-study variability. A comprehensive preprocessing routine was then applied to each dataset. The impute package was used to supplement missing data (Troyanskaya et al. 2001). Next, the normalize Between Arrays function in the limma package was used to normalize gene expression. DEGs in each dataset were identified using the limma package in the R computing environment, determined by an absolute log2 fold change (| Log 2 FC|) greater than 0 and an adjusted p-value less than 0.05 (Ritchie et al. 2015). Volcano plots were generated using ggplot2 in R. Finally, the Venn Diagram, a web-based tool, was employed to identify key genes from upregulated DEGs and subcluster-specific genes of neurotoxic astrocytes. Interactions among key genes and neurotoxic astrocyte-specific genes overlapping with upregulated DEGs were investigated using STRING (selecting high confidence 0.7) for protein–protein interactions (PPI) network analysis, and the results were visualized in Cytoscape (version 3.10.1).

### Clinical Correlation Analysis and Gene Set Enrichment Analysis

We enrolled the dataset GSE106241 and compared the expression level of key genes in different disease stages. We investigated their associations with alpha-secretase activity, gamma-secretase activity, β-secretase activity, braak stage, and Aβ_42_ levels in samples from GSE106241 using the Spearman correlation analysis. The Agora’s Gene Comparison Tool on Synapse.org was used for key gene validation. Next, Gene Set Enrichment Analysis (GSEA) was conducted on the GSE48350, GSE33000 and GSE106241 datasets to identify Biological Process (BP) and GO terms associated with the key genes. The analysis was performed using the clusterProfiler package in R. As reference gene sets, the c5.bp.v7.0.symbols.gmt datasets from the MsigDB Version 6.2 database were utilized. Gene sets that showed a significantly enriched result with an adjusted p-value < 0.05 after 1,000 permutations were considered as significant findings.

### Animals

The 5 × FAD model mice and wild-type (wt) mice, six in each group, were utilized for the study. The care, feeding, and handling of all animals were performed in adherence to the Guide for the Care and Use of Laboratory Animals from Chongqing Medical University. The experimental design was conducted randomly and in a double-blind fashion with adult mice. All experiments were designed to minimize animal suffering and to limit the number of animals used.

### Primary Perinatal Astrocyte Culture and Isolation

Primary astrocyte cultures were prepared from the cerebral neocortex of P0-P3 neonatal mice, as previously described with slight modifications (Huang et al. 2016). Briefly, brain tissue was isolated from the skull, meninges were gently removed, and the tissue was minced and passed through a 70 μm filter to generate a single-cell suspension. The mixed glial cells were then cultivated in T75 flasks with 10% heat-inactivated fetal bovine serum (CTCC-002–071,Meisen, Zhejiang, China) and 1% penicillin/streptomycin (BL505A, Biosharp, Hefei, China) in DMEM (21,068,028, Gibco, Massachusetts, USA) for 12–14 days.

### Preparation of Aggregated Aβ_42_

The toxic oligomers of Aβ_42_ (107,761–42-2, Sigma, USA). were prepared as described (Stine et al. 2011). Briefly, peptide was dissolved in hexafluoroisopropanol, dried under vacuum, and stored at − 20 °C. Immediately prior to use, the peptide residue was reconstituted in DMEM media to make a stock solution at 0.1 mM and incubated at 4 °C for 24 h to form diffusible oligomers. Aβ_42_ oligomers at a final concentration of 2 μM were assayed for astrocytes.

### Transfection

WW domain-containing transcription regulator 1 (*WWTR1*) overexpression plasmid was constructed by TSINGKE (Wuhan, China). Primary astrocytes were transfected with *WWTR1* overexpression plasmids. Cells were cultured at 60%-70% confluence in 10 cm plates and were transfected using Lipofectamine 3000 (L3000001, Invitrogen, Boston, USA). The empty vector plasmids were transfected in the same way. At 48 h post-transfection, the efficacy of transfection was identified by RT-PCR.

### Western Blotting

Western blotting (WB) was performed according to the description mentioned earlier. Brain tissues from 9-month-old wt and 5 × FAD mice were collected. Tissue and cell proteins were extracted using RIPA lysis buffer (P0013B, Beyotime, Shanghai, China) and phenylmethanesulfonyl fluoride (PMSF, ST506, Beyotime, Shanghai, China). After centrifugation at 16,000 rpm (4 °C), the supernatant was collected and stored at − 80 °C. The protein concentration was determined using the BCA protein assay kit (P0010, Beyotime, Shanghai, China). The supernatant samples were separated on a 10% SDS-PAGE gel and transferred onto a 0.22 μm polyvinylidene fluoride (PVDF) membrane (GVHP29325, Millipore, Billerica, MA, USA). The membrane was blocked with 5% skim milk in tris-buffered saline with Tween-20 (TBST) at room temperature for 2 h. Then, it was incubated with the primary antibody (diluted in TBST buffer at 1:3000) specific for WWTR1 (66,500–1-Ig, Proteintech, Wuhan, China, RRID: AB_2881864) overnight at 4 °C. The next day, the membrane was washed and then incubated with the PBST secondary antibody (1:10,000, SA00001-1, Proteintech, Wuhan, China, RRID: AB_2722565) at room temperature for 1 h. The bands were visualized using an enhanced chemiluminescence reagent (WBKLS0100, Thermo, Marina, CA, USA) and an image analysis system (Bio-Rad, USA). The membrane was then stripped using a stripping buffer (P0025, Beyotime, Shanghai, China), re-blocked and incubated with the HRP-conjugated β-actin antibody (1:10,000, HRP-60008, Proteintech, Wuhan, China, RRID: AB_2819183). Relative quantification analysis was performed using ImageJ software and reference proteins.

### Immunostaining

The procedure involves fixing brain slices with cold 4% paraformaldehyde, followed by washing and permeabilization using a 0.5% Triton X-100 PBS solution, and blocking with 5% BSA. Subsequently, the samples are incubated with specific antibodies, including mouse anti-WWTR1 (1:500, 66,500–1-Ig, Proteintech, Wuhan, China, RRID: AB_2881864), rabbit anti-GFAP (1:500, 16,825–1-AP, Proteintech, Wuhan, China, RRID: AB_2109646), rabbit anti-NeuN (1:500, 26,975–1-AP, Proteintech, Wuhan, China, RRID: AB_2880708), and rabbit anti-Iba1 (1:500, 10,904–1-AP, Proteintech, Wuhan, China, RRID: AB_2224377). The secondary antibodies used in this experiment are DyLight 488 Goat Anti-Mouse IgG (1:500, A23210, Abbkine Inc., Wuhan, China) and Dylight 594 Goat Anti-Rabbit IgG (1:500, A23420, Abbkine Inc., Wuhan, China). DAPI (1:1000, C1002, Beyotime, Shanghai, China) is used for nuclear visualization. The primary antibodies used in this study were validated and widely used in previous publications, including WWTR1 (Hu et al. 2021), GFAP (Boivin et al. 2021), IBA1(Xu et al. 2021a), NEUN(Li et al. 2021), HRP-conjugated β-actin (Shiraishi et al. 2023). To clarify the cellular localization of WWTR1, three random images were taken at a 60X magnification and then analyzed using ImageJ software. To analyze the differences in WWTR1 expression within the brain sections, the stained samples were scanned by using an automatic slide scanner (VS200, Olympus, Tokyo, Japan). The digital images were analyzed using the Visiopharm software module (Visiopharm, Hørsholm, Denmark). The whole cortical and hippocampal regions of the brain sections were selected separately for this purpose. The software automatically identified all nuclei expressing GFAP, and these were subsequently auto-labeled. Nuclei co-expressing GFAP and WWTR1 were marked in red, while those expressing GFAP but not WWTR1 were marked in white. The software's AI capabilities were utilized to calculate the proportion of WWTR1-positive cells in the WT and 5 × FAD groups.

### Quantitative Real-­Time PCR (qRT-PCR)

Total RNA from the mouse cortex was extracted using an RNA-Easy™ Isolation Reagent Vazyme Cat (RC112-01, Vazyme, Nanjing, China). HiScript II Q RT SuperMix for qPCR (+ gDNA wiper) (R223-01,Vazyme, Nanjing, China) was used for reverse transcription. Quantitative PCR was performed using Universal SYBR Green Fast qPCR Mix was acquired from ABclonal (RK21203, Wuhan, China). The fold change of gene expression was calculated using the 2^−ΔΔCt^ method. The primers were synthesized by tsingke Biotech and presented in Table S1.

### Statistical Analysis

Sample size calculations utilized the formula E = total number of animals per group—number of groups. Each experiment ensured a value of E > 10(Charan and Kantharia 2013). Following previous research guidelines and setting a significance criterion at *α* = 0.05 and power at 0.80, the minimum required sample size with this effect size for a Wilcoxon-Mann Whitney test between two groups was determined to be *N* = 3 (Reid et al. 2023). In order to bolster data robustness, 5–6 animals were included in each experimental group. According to existing literature (Reid et al. 2023), the least sample size required is *N* = 4. Nevertheless, factoring in the diversity in effect size and data, it is postulated that a sample size of *N* = 6–7 per group would be adequate. The absence of a priori sample size calculation emerged as a potential limitation in this method. Parametric data was presented as mean ± SD and non-parametric data as medians ± interquartile range. Normality was evaluated using the Shapiro–Wilk test and variance homogeneity was assessed by the Levene test. For comparing two groups, when the data was normally distributed and exhibited homogeneity of variances, we utilized a two-tailed unpaired t-test. In cases where the data was normally distributed, but variances were not equal, Welch's t-test was employed. All statistical analyses were conducted using GraphPad Prism software, with p-values of < 0.05 deemed statistically significant. To preserve the objectivity of the research, the authors were blinded to the experimental protocol and did not have access to statistical calculations during the execution of experiments. The detailed information on statistical tests was in the Table S2-5.

## Results

### Integration of Multiple AD Single-Nucleus RNA Sequencing Datasets and Cell Type Identification

**Fig. 1:**
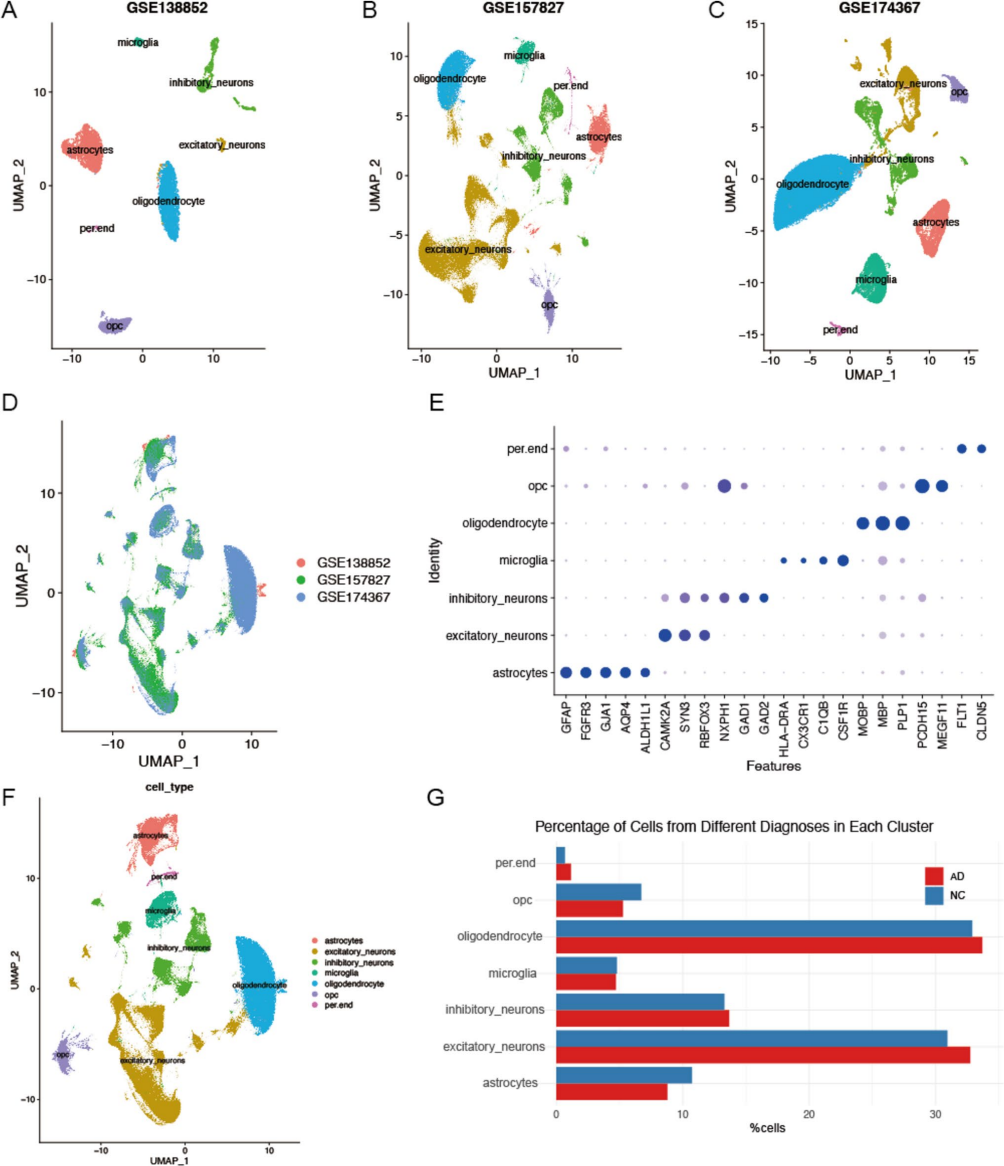
Integration of AD snRNA-seq data and clustering of cells. **A** UMAP of GSE138852. **B** UMAP of GSE157827. **C** UMAP of GSE174367. **D** UMAP distribution of cells annotated by cohorts. 30 AD patient samples, including a total of 210,654 cells were included for down-stream analysis. Cells from different cohorts were evenly merged. **E** Dot plot depicting selected differentially expressed genes for each cluster. Dot size corresponds to the percentage of nuclei expressing the gene in each cluster; color represents the average gene expression level. **F** UMAP distribution of cells colored by the major cell types. **G** Histogram showing the percentage of cells from different cell types in AD and NC

Given the inherent limitations of individual datasets and the risk of biased outcomes, integrating data from multiple studies or experiments is crucial for robust results (Wang et al. 2022). In the current research, we have amalgamated three available snRNA-seq datasets of AD, as detailed in Table 1, following the stringent selection criteria outlined in our Methods section. Then, we performed UMAP analysis on each dataset separately and defined the cell populations for the three datasets (Fig. 1A-C).

After meticulous quality control and batch-effect correction using Harmony, the three datasets were integrated into one unified analysis, encompassing 210,654 cells for downstream analysis (Fig. 1D). This integrated approach significantly enhances the diversity and volume of the data, providing a comprehensive basis for our subsequent analysis. Based on previous studies (Chen et al. 2023; Darmanis et al. 2015), the cells were categorized into seven major cell types using the Seurat “FindAllMarkers” function. These types included astrocytes (*n* = 20,276) identified by *GFAP, FGFR3, GJA1, AQP4, ALDH1L1*; excitatory neurons (*n *= 67,301) by *SYN3, RBFOX3, CAMK2A;* inhibitory neurons (*n* = 28,450) by *ERBB4, NXPH1, GAD1, GAD2*; microglia (*n* = 9991) by *HLA-DRA, CX3CR1, C1QB, CSF1R*; oligodendrocytes (*n* = 70,257) by *MOBP, MBP, PLP1*; OPCs (*n* = 12,413) by *PCDH15, MEGF11*; and endothelial cells (*n* = 1966) by *FLT1, CLDN5*. Assessing the distribution of cells from different cohorts in the integrated data showed that cells across cohorts were evenly integrated, and each major cell lineage contained cells from different cohorts, indicating that the data integration across cohorts was successful (Fig. 1E-F). Cell population analysis revealed considerable differences between AD participants and non-AD controls, with an elevation in excitatory neurons and oligodendrocytes in AD participants (Fig. 1G).

### Cell subtyping of Astrocytes Reveals Neurotoxic Astrocyte Subcluster

**Fig. 2:**
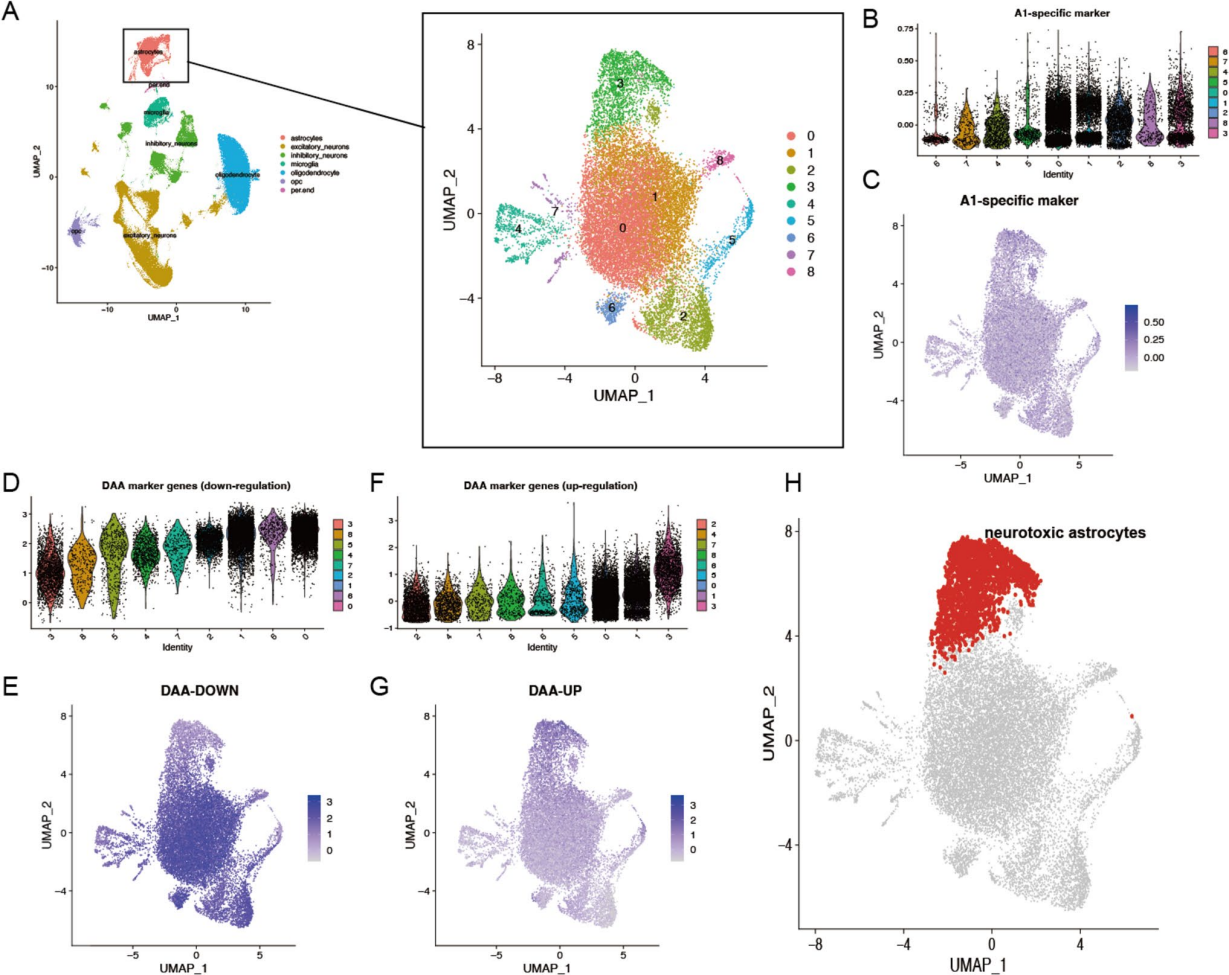
Reclustering of astrocytes and neurotoxic astrocyte subcluster identification. **A** UMAP plot showing the reclustering of astrocytes. **B** Violin plot represents the distribution of A1-specific transcript signature scores across subclusters of astrocytes. **C** UMAP illustration of the distribution of a1-specific transcript expression. Cluster 3 exhibits the highest distribution. **D** Violin plot of downregulated DAAs marker across subclusters of astrocytes. Cluster 3 exhibits the lowest distribution. **E** UMAP representation of downregulated DAAs marker expression. **F** Violin plot of upregulated DAAs marker across subclusters of astrocytes. Cluster 3 exhibits the highest distribution. **G** UMAP representation of upregulated DAAs marker expression. **H** Cluster 3 astrocytes are identified as neurotoxic astrocytes

As astrocytes have been found to play a complex and dual role in AD, understanding the biological characteristics of neurotoxic astrocytes and identifying their specific markers are of great importance. Novel biomarkers of neurotoxic astrocytes could help the development of therapeutic strategies and potentially halt or slow the progression of AD (Price et al. 2021). By integrating A1 and DAAs markers, harmful astrocyte clusters in AD can be identified. Therefore, astrocytes were isolated from the integrated data for re-clustering, which revealed nine subclusters (Fig. 2A). To characterize the specific subpopulation of A1 astrocytes, A1-specific transcriptional markers were employed, including *GGTA1P, AMIGO2, FBLN5, FKBP5, GBP2, PSMB8, SERPING1,* and *SRGN* (Liddelow et al. 2017). By calculating signature scores using the AddModuleScore method, the A1 markers were found predominantly expressed in cluster 3 (Fig. 2B-C). Next, a subcluster of astrocytes, called DAAs were defined using downregulated markers including *SLC1A2, SLC1A3, GLUL, NRXN1, CADM2, PTN, GPC5* and upregulated markers *GFAP, CD44, HSPB1, TNC* (Xu et al. 2021b). Interestingly, cluster 3 exhibited the lowest expression of downregulated markers (Fig. 2D-E) and the highest expression of the upregulated markers (Fig. 2F-G). Therefore, the DAAs subpopulation was also concentrated within cluster 3. Finally, cluster 3 emerged as a cell population highly associated with disease characteristics, defining it as the neurotoxic astrocyte subpopulation of interest for focused investigation (Fig. 2H).

### Enrichment Analysis and Identification of Key Genes in Neurotoxic Astrocytes

**Fig. 3:**
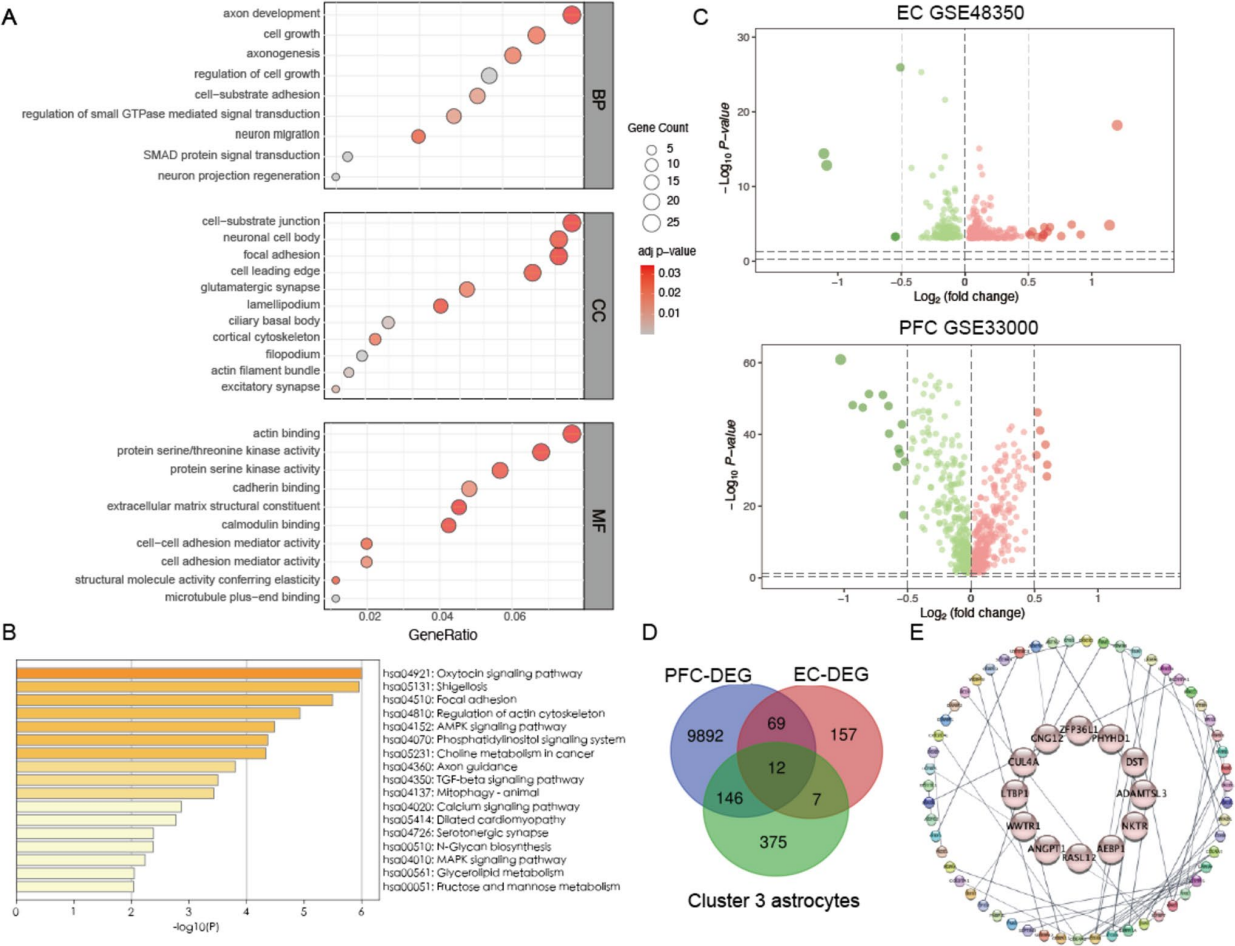
Enrichment analysis and identification of key genes in neurotoxic astrocytes. **A** the GO analysis of genes in cluster 3 neurotoxic astrocytes. **B** the KEGG pathway analysis of cluster 3 neurotoxic astrocytes. **C** The volcano plot of the genes in the PFC and EC. **D** Venn diagram analysis of the intersection between upregulated differential genes in bulk data and cluster 3 astrocytes. **E** PPI network of the 12 key genes and neurotoxic astrocyte-specific genes overlapping with upregulated DEGs

The biological characteristics of neurotoxic astrocytes associated with AD are not yet fully understood and further exploration is crucial. Here, gene ontology (GO) and KEGG enrichment analysis were performed on astrocyte cluster 3 to explore the involved biological process of neurotoxic astrocytes. The analysis of GO annotations revealed that biological processes (BP) in neurotoxic astrocytes are primarily associated with cell growth, axon development, axonogenesis, neuron migration, and neuron projection regeneration. For cellular component (CC) enrichment analysis, the results showed that neurotoxic astrocytes significantly took part in neuronal cell body, glutamatergic synapse, cortical cytoskeleton and excitatory synapse. For molecular function (MF) analysis, neurotoxic astrocytes-associated genes are mainly enriched in protein serine/threonine kinase activity, cadherin binding and calmodulin binding (Fig. 3A). The KEGG pathway analysis showed that the neurotoxic astrocytes-associated genes were significantly enriched in the axon guidance, N-Glycan biosynthesis, fructose and mannose metabolism, glycerolipid metabolism and calcium signaling pathway, which are crucial for astrocytes function and neuron survival (Fig. 3B).

To determine the novel markers of neurotoxic astrocytes, we further enrolled two independent AD bulk RNA-seq datasets to perform an integrative analysis. Differential gene analysis was performed on the PFC transcriptome data from datasets GSE33000 and EC datasets of GSE48350 (Fig. 3C). The significantly upregulated differentially expressed genes in AD intersected with the neurotoxic astrocyte cell population, resulting in the identification of twelve key genes, including *GNG12, ZFP36L1, AEBP1, WWTR1, PHYHD1, RASL12, DST, CUL4A, ADAMTSL3, LTBP1, NKTR, ANGPT1* (Fig. 3D, Table 2). PPI analysis uncovered an interaction network between these 12 key genes and neurotoxic astrocyte-specific genes overlapping with upregulated DEGs (Fig. 3E).

**Table 2:** The list of 12 markers of AD associated neurotoxic astrocytes

UniProt ID	Gene Symbol	Gene Name	Function
Q5VZM2	GNG12	protein G-protein subunit gamma 12	G-protein signaling modulator
Q07352	ZFP36L1	Zinc finger protein 36-like 1	mRNA destabilization
Q6ZN18	AEBP1	Adipocyte enhancer-binding protein 1	involved in adipogenesis and regulation of gene expression
Q9HAW4	WWTR1	WW domain-containing transcription regulator protein 1	cellular proliferation and tissue development
Q8N0X4	PHYHD1	Phytanoyl-CoA dioxygenase domain-containing protein 1	metabolism of phytanic acid
Q8NCE0	RASL12	Ras-like protein family member 12	cytoskeletal organization, and membrane trafficking
Q92997	DST	Dystonin	plays a role in cytoskeletal organization and cell adhesion
Q13619	CUL4A	Cullin-4A	part of the E3 ubiquitin ligase complex involved in protein degradation and cell cycle regulation
Q8NBJ5	ADAMTSL3	ADAMTS-like protein 3	extracellular matrix organization and tissue development
P22003	LTBP1	Latent transforming growth factor-beta-binding protein 1	regulating the bioavailability and activation of TGF-beta
Q8TDX7	NKTR	Natriuretic peptide receptor	involved in the regulation of blood pressure and fluid balance
Q15389	ANGPT1	Angiopoietin-1	plays a role in angiogenesis and vascular development

### Functional Analysis and Expression Validation of Key Genes

**Fig. 4:**
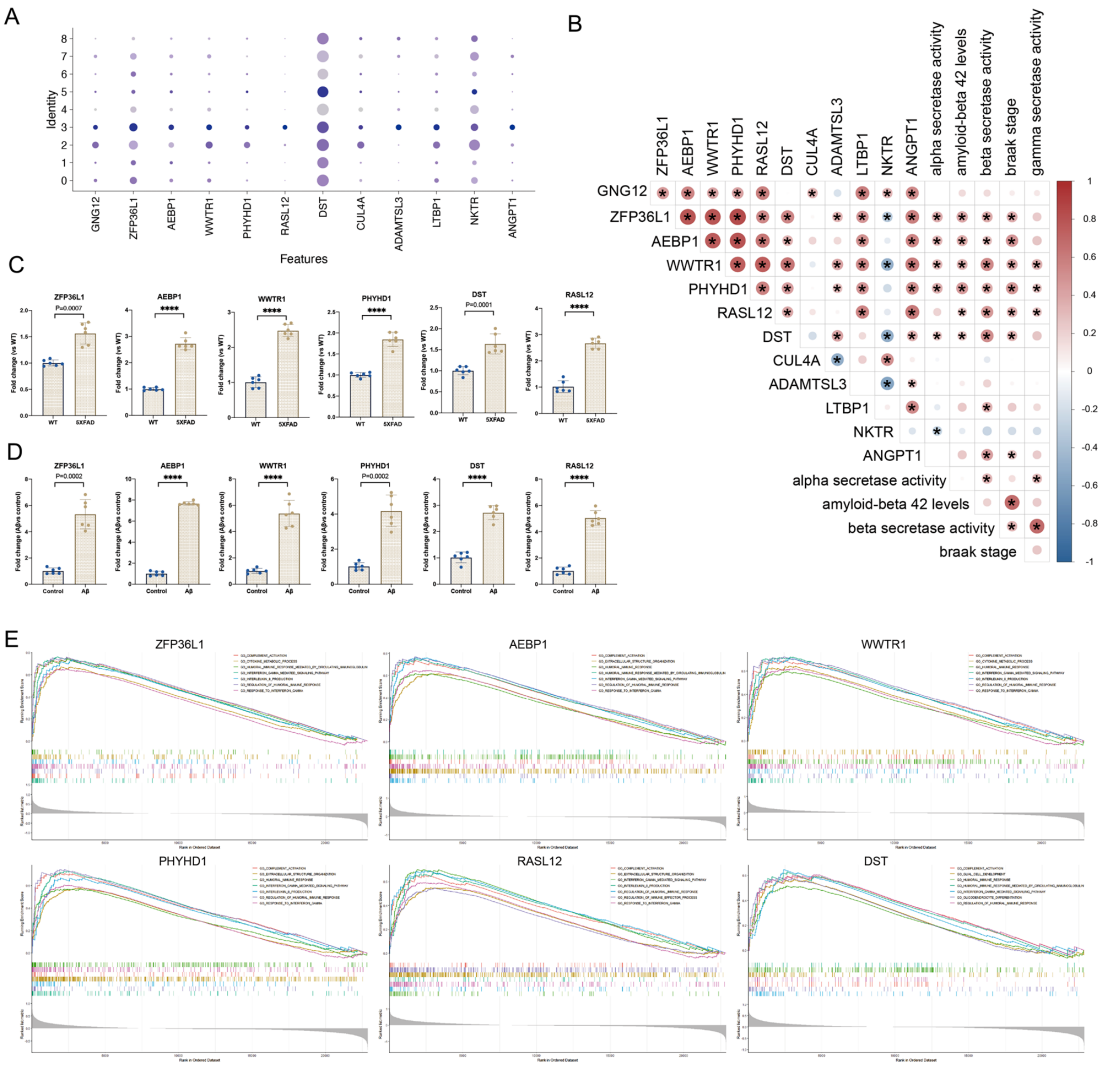
Clinical correlation analysis, functional analysis, and expression verification of key genes. **A** Dot plot depicting 12 key genes expression in different astrocytes cluster. **B** Correlation heatmap illustrating the analysis of the relationship between 12 key genes and clinical disease severity markers based on GSE106241. **C** RT-qPCR validation of the expression of six clinical severity-related genes in the brain tissue of 5 × FAD mice (*n* = 6 per group). **D** RT-qPCR validation of the expression of six clinical severity-related genes in primary astrocytes treated with Aβ (*n* = 6 per group). **E** GSEA of six clinical severity-related genes based on GSE48350. (****p < 0.0001)

Firstly, the expression of the 12 key genes was validated, and they were found to be significantly upregulated in neurotoxic astrocytes clusters (Fig. 4A). To explore the clinical-pathological relevance of these key genes with AD, an independent dataset, GSE106241, was incorporated to analyze the correlation between the expression levels of the key genes and various AD pathological indicators, including alpha-secretase activity, Aβ_42_ levels, beta-secretase activity, and Braak stage, as well as gamma-secretase activity. *WWTR1* and *PHYHD1* are significantly correlated with all five AD pathological indicators. *ZFP36L1, AEBP1* and *DST* were associated with alpha-secretase activity, Aβ_42_ levels, beta-secretase activity and Braak stage. *RASL12* was associated with Aβ42 levels, beta-secretase activity, Braak stage, and gamma-secretase activity (Fig. 4B). The six key genes were validated using Agora’s Gene Comparison Tool on Synapse.org and were significantly upregulated in various brain regions with notable increases in risk coefficients (Figure S1).

Next, the expression levels of the six disease-associated genes *ZFP36L1*, *AEBP1*, *WWTR1*, *PHYHD1*, *DST*, and *RASL12* were validated through qPCR, with significant upregulation observed in the brain tissue of 5 × FAD mice and in primary astrocytes treated with Aβ42 (Fig. 4C). Additionally, a marked increase in their expression was observed in primary astrocytes subjected to Aβ treatment (Fig. 4D). These results underscore the potential role of these genes in astrocyte-mediated responses to Aβ pathology.

Lastly, GSEA was conducted on each of the six disease-associated genes based on their expression profiles in three datasets to investigate their biological functions (Fig. 4E, Figures S2-3). All of the six key genes were found to be enriched in pathways related to humoral immune response, complement activation, and cytokine metabolic processes in each dataset. These results suggest that these genes may play pivotal roles in modulating immune responses and cytokine signaling pathways, underscoring their potential significance in the disease's pathophysiology. Additionally, GSEA analysis of GSE33000 revealed *ZFP36L1, PHYHD1*, and *RASL12* were associated with astrocyte development, and *RASL12* was also linked to amyloid fibril formation. In GSE106241, *PHYHD1* was found to be associated with amyloid-beta clearance. These findings further confirm the crucial roles of these six genes in astrocytes and their relevance to AD.

### Functional Validation of WWTR1 in 5 × FAD Mice and Astrocytes

**Fig. 5:**
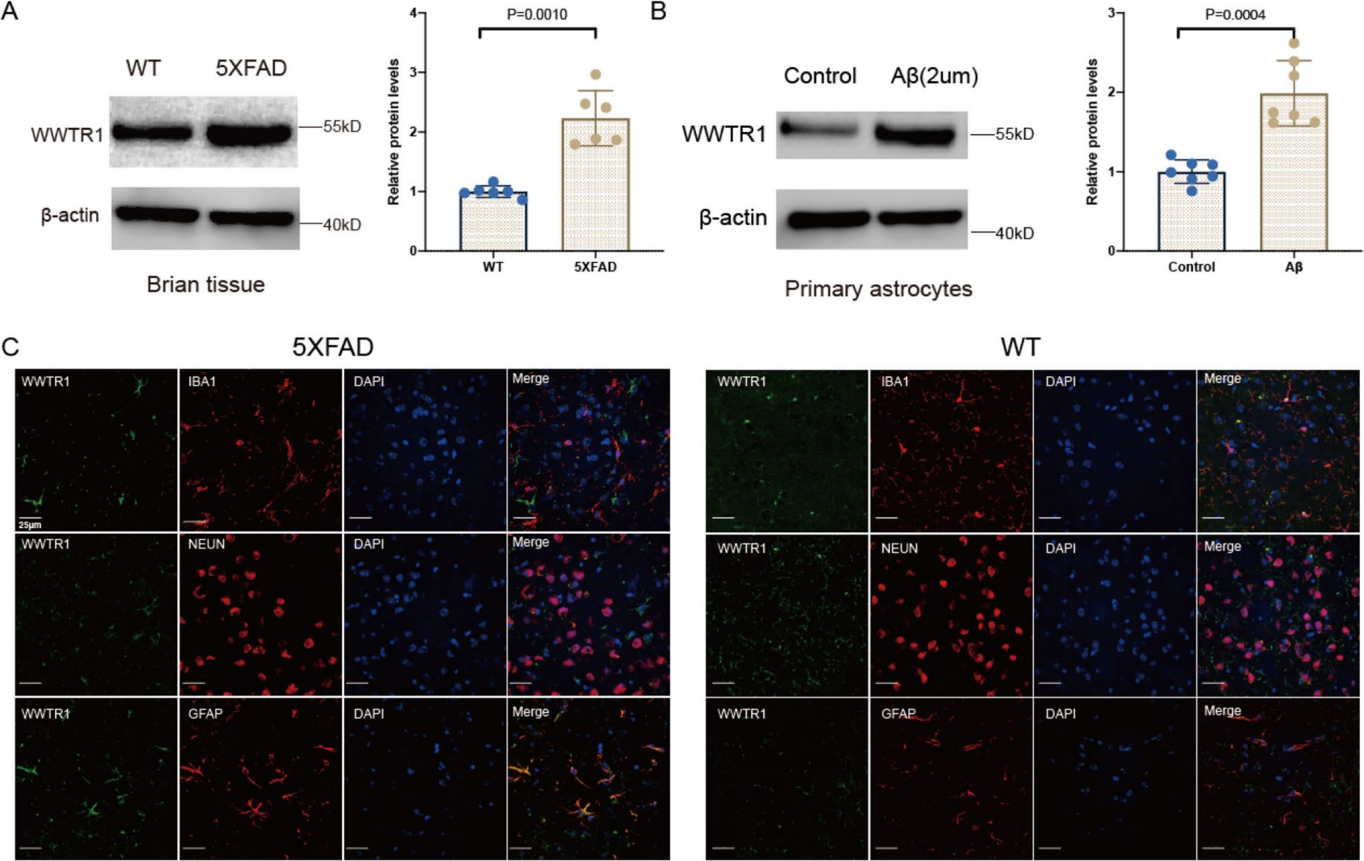
Verification of WWTR1 expression and localization in 5 × FAD mice brain. **A** Western blot analysis confirming the expression changes of WWTR1 in 5 × FAD mice brain (*n* = 6 per group). **B** Western blot analysis confirming the expression changes of WWTR1 in Aβ treated primary astrocytes and control astrocytes (*n* = 7 per group). **C** Immunofluorescence images indicate that WWTR1 is not colocalized with IBA1 and NEUN, but colocalizes with GFAP in the hippocampus and cortex of the 5 × FAD and WT mice. (****p < 0.0001)

Our previous research has identified *WWTR1* as a critical gene of AD (Yu et al. 2021). Therefore, the expression and localization of *WWTR1* and its role in astrocytes need to be further confirmed. Firstly, the expression of WWTR1 was validated using 5 × FAD mice. WB analysis revealed a significant upregulation of WWTR1 expression in the brain tissue of 5 × FAD mice (Fig. 5A, Figure S4). Next, primary astrocytes were treated with 2 µM Aβ for one day, after which cell proteins were extracted. The expression of WWTR1 was significantly increased after Aβ treatment (Fig. 5B, Figure S4). To clarify the cellular localization of WWTR1, immunofluorescent staining was performed on brain sections. WWTR1 did not co-localize with the microglial marker IBA1 or the neuronal marker NeuN in both 5 × FAD and wild-type (WT) mice. However, WWTR1 exhibited significant co-localization with the astrocyte marker GFAP (Fig. 5C). Thus, WWTR1 is primarily expressed in astrocytes.

**Fig. 6:**
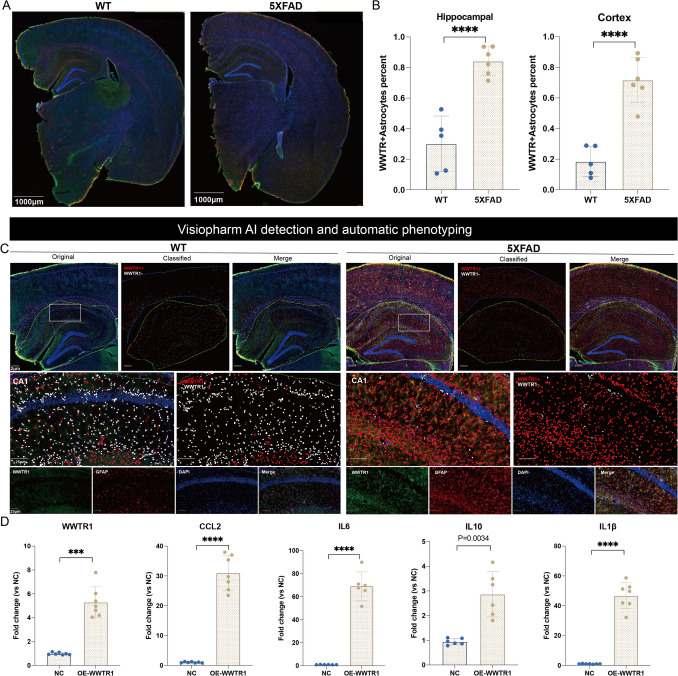
Significant increase in WWTR1 expression in astrocytes of 5 × FAD mice and its role in driving pro-inflammatory responses. **A** Representative hemisphere stitches of WT and 5 × FAD (WWTR1 in green, GFAP in red, DAPI in blue). Scale bar = 1000 μm. **B** In GFAP + astrocytes of hippocampal and cortex tissue from 5 × FAD mice, wwtr1 expression is significantly increased. (wt, *n* = 5; 5 × FAD, *n* = 6). All stained slides were quantified with Visiopharm Integrator System software. **C** Representative high magnification images of Immunofluorescence staining in the CA1 of wt and 5 × FAD mice. Scale bar = 25 μm; Segmentation of the digital image using Visiopharm Image Analysis Software where red pixels designate WWTR1 + astrocytes and white pixels define WWTR- astrocytes. **D** RT-qPCR verification of overexpression efficiency of WWTR1 and expression of inflammatory factors in primary astrocytes (WWTR1, CCL2, IL1β: *n* = 7 per group; IL6, IL10: *n* = 6 per group; ***p = 0.0001, ****p < 0.0001)

To investigate the expression of WWTR1 in 5 × FAD astrocytes, further analysis was performed on the immunofluorescence of 5 × FAD and WT mice (Fig. 6A). Through automatic analysis with Visiopharm, the proportion of WWTR1 + astrocytes of both the hippocampus and cortex was found to significantly increase among the GFAP + astrocytes in 5 × FAD mice (Fig. 6B-C). The results indicate that the expression of WWTR1 in 5 × FAD astrocytes significantly increased. These findings provide evidence that WWTR1 may serve as a marker for neurotoxic astrocytes in the brain tissue of 5 × FAD mice, demonstrating its potential involvement in AD pathology.

The pro-inflammatory role of *WWTR1* in astrocytes was further verified. Primary astrocytes were treated with a *WWTR1* overexpression plasmid, while the negative control (NC) group was treated with empty vector plasmids. The expression of inflammatory factors was verified by qRT-PCR. The results showed that when *WWTR1* was increased by 4–6 times, the inflammatory factors *IL1β, IL6, IL10,* and *CCL2* all significantly increased (Fig. 6D). Therefore, *WWTR1* might exacerbate AD pathology through its pro-inflammatory effects.

## Discussion

In this study, snRNA-seq data from human brain tissue are integrated for holistic analysis, and the biological characteristics of the neurotoxic astrocyte subcluster associated with AD are also described. By combining bulk data, *ZEP36L, AEBP1, WWTR1, PHYHD1, DST* and *RASL12* are identified as novel markers of neurotoxic astrocytes which are increased in both 5 × FAD mice and primary astrocytes with Aβ treatment. Finally, WWTR1 is found to be closely related to neurotoxic astrocytes and the progression of AD pathology. WWTR1 is primarily expressed in astrocytes and significantly increased in 5 × FAD mouse astrocytes. Moreover, overexpression of WWTR1 significantly drives the pro-inflammatory action of astrocytes. Therefore, WWTR1 may be a unique marker and an important target of neurotoxic astrocytes in AD.

SnRNA-seq data analysis has played a significant role in advancing our understanding of AD and has contributed to important discoveries in recent years (Morabito et al. 2021; Miller et al. 2022). By examining individual cells within brain tissues, snRNA-seq enables the identification of cell subtypes, characterization of their gene expression profiles, and exploration of cellular heterogeneity in AD (Mathys et al. 2019). For example, Yijing Su et al. revealed the diversity of glia differentiated and identified dysregulated genes and pathological processes in specific glial subpopulations in AD (Su et al. 2022). However, individual snRNA-seq datasets may present several limitations. The sample size of individual datasets was relatively small and the samples all came from a single center, resulting in regional biases and sampling biases. This can lead to a less comprehensive representation of the overall population or phenomenon under study. The limitation can subsequently affect the generalizability of the results. The integration of multiple datasets is an effective strategy, which can increase the sample size and the number of cells, thereby enhancing the credibility and reliability of the results (Wang et al. 2022). Therefore, in our current study, we integrate snRNA-seq data from three human brain databases for further analysis. The data is dimensionally reduced, and cell clusters are classified based on characteristic markers from multiple cells, consequently reducing errors and enhancing the credibility of the results.

In the current study, the snRNA-seq datasets included samples from the PFC and the EC. The PFC, a substantial part of the brain's cortex, accounts for a significant portion of the cerebral cortex and is commonly considered one of the largest regions in the human brain (Carlén 2017). The EC, though smaller, plays a vital role in memory formation and spatial memory. It is a part of the parahippocampal gyrus and is crucial for cognitive functions and its relation to neurodegenerative diseases such as AD (Takehara-Nishiuchi 2014; Olajide et al. 2021). Thus, snRNA-seq data derived from these two brain regions are of significant research importance and representational value, especially in the biomarkers exploration.

Recent research into AD mechanisms has highlighted the limitations of focusing exclusively on neurons. The role of neurotoxic astrocytes in AD is garnering increasing interest (Grimaldi et al. 2019). Further, as the understanding of reactive astroglia deepens, it has become evident that identifying reactive astrocytes necessitates multiple markers, moving beyond the sole reliance on GFAP expression (Escartin et al. 2019). Therefore, the current study analyzed 20,276 cells and classified them into 9 distinct clusters to explore the functional characteristics of astrocytes in AD. In recent years, some researchers have divided astrocytes into A1 and A2 types. The A1 astrocytes exhibit a gene expression pattern associated with detrimental effects on neurons, such as synaptic loss and neuroinflammation. In contrast, A2 astrocytes are typically considered neuroprotective (Liddelow et al. 2017). However, defining AD's cell population solely based on A1 astrocytes has its limitations. By incorporating A1-specific transcripts and DAA markers, cluster 3 is identified as neurotoxic astrocytes and their transcriptional features are explored. GO enrichment analysis reveals that this subpopulation is involved in cell growth, axon development, neuron migration, glutamatergic synapse, cortical cytoskeleton, and excitatory synapse. In the AD brain, the presence of amyloid-beta (Aβ) impairs the efficient uptake of glutamate by astrocytes, leading to neuronal swelling, compromised membrane integrity, and ultimately excitotoxicity (Conway 2020; Andersen et al. 2021). Our enrichment analyses provide deeper insights into the potential molecular mechanisms and interactions of neurotoxic astrocytes in AD. They underscore the importance of specific cellular processes, molecular functions, and protein interactions that may contribute to the astrocytes' neurotoxic effects in disease progression.

SnRNA-seq technology enhances the precision of cellular differentiation analysis and enriches our understanding of individual cell functions. However, it also somehow generally has different levels of technical variability (Myers et al. 2020). Integrating single-nucleus and bulk sequencing data can provide a synergistic approach that enhances the reliability, accuracy, and comprehensiveness of the analyses (Sun et al. 2022). Therefore, an integrative analysis of bulk RNA-seq and SnRNA-seq is conducted and 12 genes associated with neurotoxic astrocytes and AD are identified. Among these genes, *ZFP36L1, AEBP1, WWTR1, PHYHD1, DST,* and *RASL12* are associated with AD pathology indicators. Their expression was significantly higher in both 5 × FAD mice and Aβ treated astrocytes. Moreover, these genes are closely related to inflammatory responses, humoral immune response, and the complement pathway in functional enrichment analysis.

Previous studies have found that neurotoxic astrocytes accelerate the progression of AD in various ways. These include influencing neurotransmitter uptake (Mahmoud et al. 2019), gliotransmitter release (Wang et al. 2019), metabolic activity (Gavillet et al. 2008), ion buffering (Tong et al. 2014), the release of cytokines, complement factors or trophic factors (Liddelow et al. 2017), phagocytosis, and the production and detoxification of reactive oxygen species (Adams and Gallo 2018). Briefly, neurotoxic astrocytes may lose their ability to regulate glutamate and phagocytose Aβ, releasing inflammatory factors which lead to synaptic dysfunction, neuronal death, and impairment of the blood–brain barrier (Lawrence et al. 2023). Therefore, the identification of markers for harmful astrocytes in AD, as well as targets for reversing their damaging effects, are of great importance. They hold significant research value and could serve as key targets for disease intervention.

Among all of the six novel markers, WWTR1 is significantly associated with Aβ pathology indicators, and our previous research has identified WWTR1 as a potential diagnostic biomarker that may contribute to the development of AD (Yu et al. 2021). In the brain of 5 × FAD mice, WWTR1 expression is significantly increased in astrocytes exhibiting elevated levels of GFAP, indicating its potential as an important marker for reactive astrocytes and involved in AD progression. Moreover, WWTR1 significantly drives the inflammatory response of astrocytes. As a transcription factor involved in the Hippo signaling pathway (Ray et al. 2022), WWTR1 participates in cell proliferation, differentiation, and tissue development (Fu et al. 2022). Previous studies indicate dysregulation of Hippo signaling, including WWTR1, has been associated with various diseases, including neurodegenerative disorders (Andl et al. 2017; Gogia et al. 2021). Previous studies have also shown that WWTR1 has been implicated in energy metabolism and inflammatory responses in tumor-related diseases (Wang et al. 2016; Zanconato et al. 2016). It has been shown to interact with nuclear factor kappa B (NF-κB) signaling, a key pathway involved in the inflammatory response (Deng et al. 2018). WWTR1 may modulate NF-κB activity and the expression of pro-inflammatory cytokines in astrocytes, potentially influencing the neurotoxic properties of these cells. However, the role of WWTR1 in neurotoxic astrocytes is still not clearly established, and further research is needed to fully elucidate its mechanisms and functional significance in AD.

## Conclusion

This study conducts an integrated analysis of both single-nucleus and bulk tissue to provide novel insights into AD pathogenesis. Notably, we have identified and validated six novel neurotoxic astrocyte biomarkers in AD. *WWTR1* is identified as a critical gene in AD astrocytes, which may provide breakthroughs into developing diagnostic markers and therapeutic targets for AD through understanding its molecular mechanisms.

## Supplementary Information

Below is the link to the electronic supplementary material.

Supplementary file1 (PDF 1479 kb)

## Data Availability

All the gene expression profiles were downloaded from the Gene Expression Omnibus (GEO) database (http://www.ncbi.nlm.nih.gov/geo/).
