# Supplementary material for: Disease-Associated Neurotoxic Astrocyte Markers in Alzheimer Disease Based on Integrative Single-Nucleus RNA Sequencing
Source: Cell Mol Neurobiol. 2024 Feb 12;44:20. doi: 10.1007/s10571-024-01453-w (PMC10861702; doi:10.1007/s10571-024-01453-w)

Disease-associated neurotoxic astrocyte markers in Alzheimer disease based on integrative single - cell sequencing analysis

Cellular and Molecular Neurobiology

Wuhan Yu[1#], Yin Li[2#], Fuxin Zhong[1], Zhangjing Deng[1], Jiani Wu[1], Weihua Yu[3], Yang Lü[1†]

†Corresponding Authors: Prof. Yang Lü,

E-mail: yanglyu@hospital.cqmu.edu.cn

Table S1. The sequences of primers.

Primer (Mouse)	Forward	Reverse
AEBP1	GTATACCTGGGCTGTGACAAGTT	CTCATCTGTCACCACACCCTTAA
DST	GAGGAGTCGGAAGTTGCTTATGA	TGCGATCTCCTGAACAGCTTTAA
PHYHD1	AAGGATTCTTCACAGCGGATGAG	CTGGGTTTGAAGTTGCTCATCTT
ZFP36L1	ATCCTAGTCCTTGCCCCGTG	GCTGGGAGTGCTGTAGTTGAG
WWTR1	CCTCAGCAACATGGACGAGA	CAGACTCCAAAGTCCCGAGG
RASL12	CTGGAAGTTAACCTGGCTATCCT	TCTTCAGCTGGTTGGGTCTTTAA
IL6	CCGGAGAGGAGACTTCACAG	TTGCCATTGCACAACTCTTT
IL10	GCTTTGGGCTTCTTGATGAG	AAGAGCCCATGAAGAGAGG
IL1β	CCTGCAGCTGGAGAGTGTGGAT	TGTGCTCTGCTTGTGAGGTGCT
CCL2	ACGTGTTGGCTCAGCCAGAT	CAGCCTACTCATTGGGATCATCT
β-actin	GAAGCTGTGCTATGTTGCTC	GAATGTAGTTTCATGGATGC

Table S2. Supplementary information for qPCR statistical data in figure 4.

Gene	WT	5xFAD	Shapiro-Wilk test	Levene's test	t(df)	pvalue
ZEP36L	1.001	1.565	>0.1000	0.015	t=6.561, df=5.789	0.0007
AEBP1	1.001	2.725	>0.1000	0.009	t=17.92, df=5.635	<0.0001
WWTR1	1.010	2.480	>0.1000	0.805	t=15.55, df=9.868	<0.0001
PHYHD1	1.001	1.849	>0.1000	0.033	t=11.58, df=6.125	<0.0001
DST	1.004	1.638	>0.1000	0.069	t=5.978, df=6.598	0.0001
RASL12	1.019	2.672	>0.1000	0.736	t=13.60, df=10	<0.0001

Gene	NC	Aβ	Shapiro-Wilk test	Levene's test	t(df)	pvalue
ZEP36L	1.021	5.334	>0.1000	0.003	t=9.265, df=5.419	0.0002
AEBP1	1.020	7.677	>0.1000	0.625	t=57.68, df=10	<0.0001
WWTR1	1.014	5.385	>0.1000	0.002	t=10.62, df=5.354	<0.0001
PHYHD1	1.018	4.179	>0.1000	0.007	t=8.334, df=5.565	0.0002
DST	1.020	2.721	>0.1000	0.545	t=12.30, df=10	<0.0001
RASL12	1.031	5.051	>0.1000	0.148	t=15.81, df=10	<0.0001

Table S3. Supplementary information for western blot statistical data in figure 5.

Sample	NC-Relative protein levels	AD-Relative protein levels	Shapiro-Wilk test	Levene's test	t(df)	pvalue
MICE-WWTR1	1.000	2.231	>0.1000	0.004	t=6.367, df=5.443	0.0010
CELL-WWWTR1	1.000	1.987	>0.1000	0.026	t=5.964, df=7.538	0.0004

Table S4. Supplementary information for visiopharm image analysis of immunofluorescence staining statistical data in figure 6.

Sample	WT	5xFAD	Shapiro-Wilk test	Levene's test	t(df)	pvalue
Hippocampal	0.302	0.843	>0.1000	0.17730	t=6.431, df=9	0.00010
Cortex	0.186	0.717	>0.1000	0.450	t=6.844, df=9	<0.0001

Table S5. Supplementary information for qpcr statistical data in figure 6.

Gene	oe-WWTR1-Avg Fold Change	NC-Avg Fold Change	Shapiro-Wilk test	Levene's test	t(df)	pvalue
WWTR1	5.290	0.983	>0.1000	<0.05	t=8.765, df=6.068	0.00010
CCL2	31.010	1.019	>0.1000	<0.05	t=13.65, df=6.014	<0.0001
IL6	89.950	1.191	>0.1000	<0.05	t=13.53, df=5.001	<0.0001
IL10	2.867	0.944	>0.1000	<0.05	t=5.088, df=5.196	0.00340
IL1β	46.830	1.010	>0.1000	<0.05	t=13.72, df=6.004	<0.0001

Figure S1. Expression of 6 Biomarker Genes in Different Brain Regions

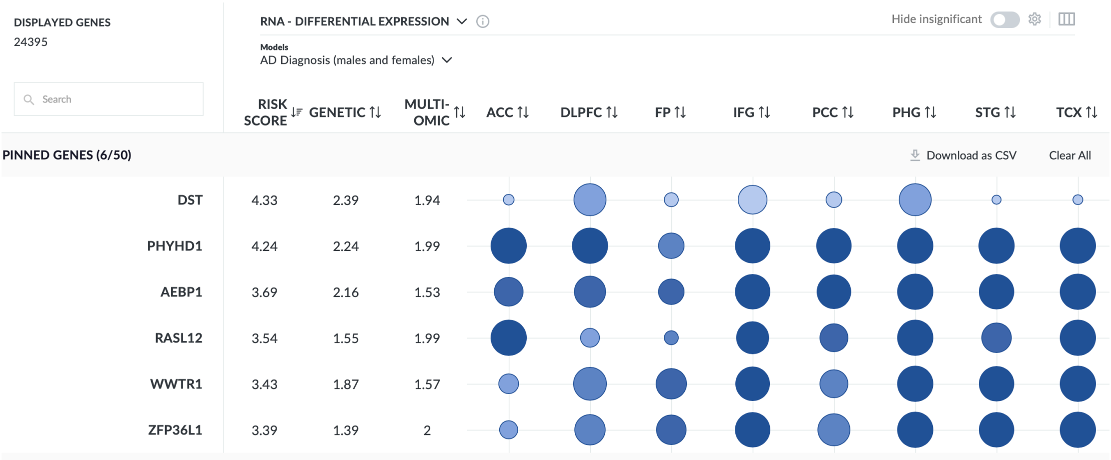

The size of the circles represents the magnitude of log2 fold change (lg2FC), and the darker the color, the smaller the p-value. Data was sourced from Synapse, and the RNA-seq data used for these analyses was generated from over 2100 samples obtained from post-mortem brains of more than 1100 individuals.

Figure S2. GSEA of six clinical severity-related genes based on GSE33000.

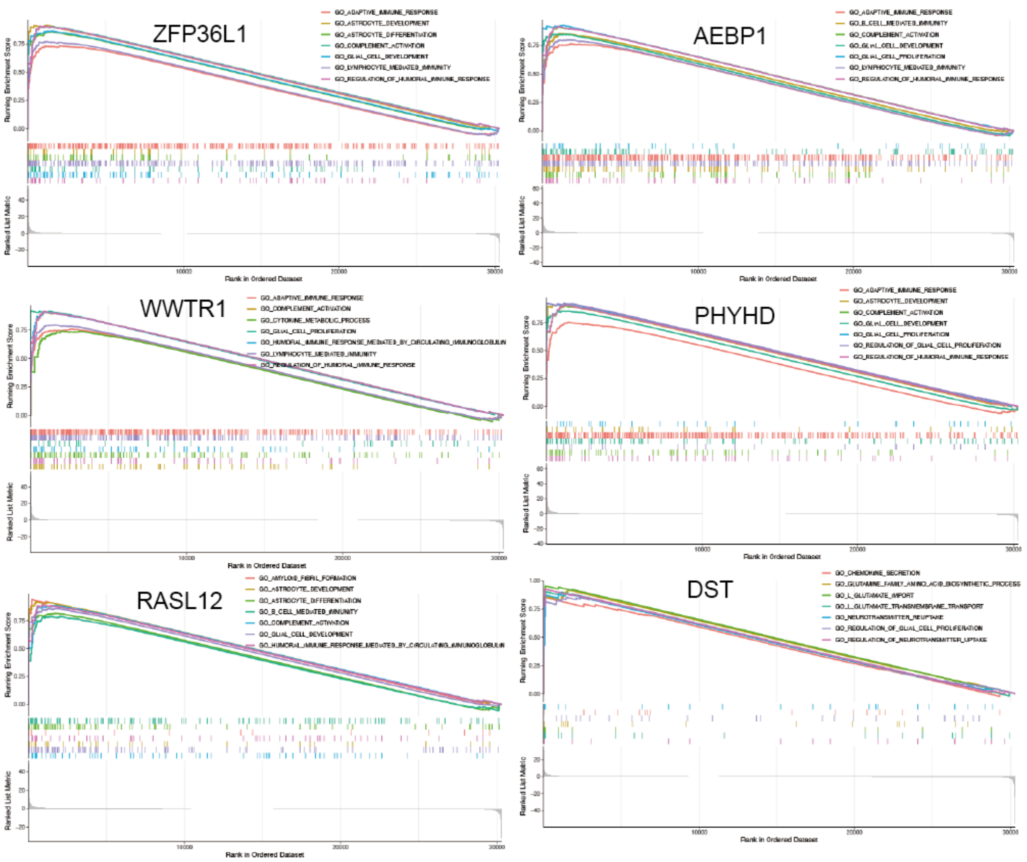

Figure S3. GSEA of six clinical severity-related genes based on GSE106241.

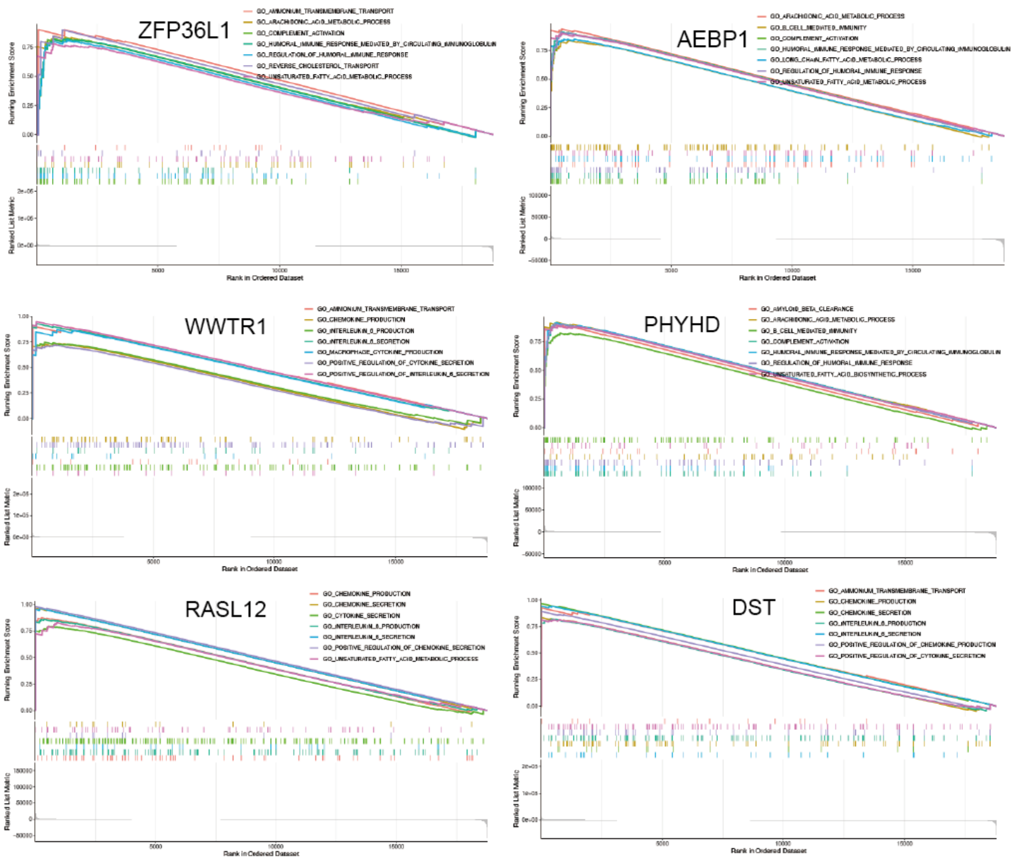

Figure S4. Original Western Blots in Figure 5.

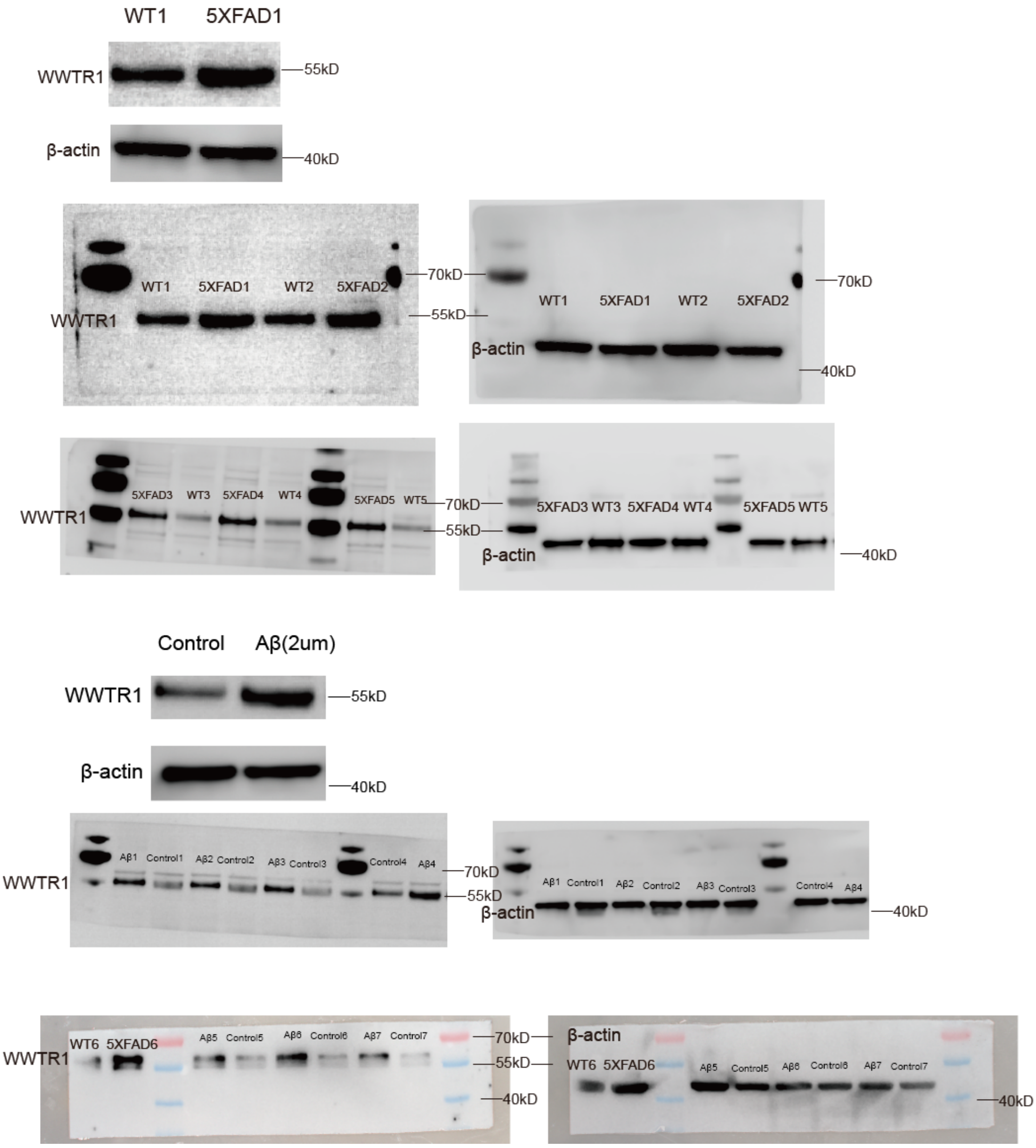

Supplement: Supplementary file 1 — Supplementary file1 (PDF 1479 kb) [file 10571_2024_1453_MOESM1_ESM.pdf]
